# Supplementary material for: Quantum Interference and Nonequilibrium Josephson Currents in Molecular Andreev Interferometers
Source: Nanomaterials (Basel). 2020 May 28;10(6):1033. doi: 10.3390/nano10061033 (PMC7420291; doi:10.3390/nano10061033)
Supplement: Supplementary File 1 [file nanomaterials-10-01033-s001.pdf]

Supplementray Information: Quantum interference and nonequilibrium Josephson current in molecular Andreev interferometers†

N. L. Plaszkó,[a] P. Rakyta,[a] J. Cserti[a], A. Kormányos[a], and C. J. Lambert[b]

[a] Dept. of Physics of Complex Systems, Eötvös Loránd University, Budapest, Pázmány P. s. 1/A, Hungary
[b] Dept. of Physics, Lancaster University, Lancaster, LA1 4YB, United Kingdom

In this supplementary material we discuss the technical details to evaluate the differential conductance. We give a closed formula to evaluate the differential conductance defined on the normal lead. We implemented our theoretical framework in the Eötvös Quantum Utilities (EQuUs) [1] software package.

S1 Theoretical background to calculate the differential conductance

In this section we give the technical details to calculate and analyze the differential conductance on the normal lead connected to an Andreev interferometer. The aim of this section is twofold. Firstly we obtain a closed formula which can be evaluated numerically. Secondly, we answer the questions raised in the discussion of the results in Fig.3 of the main text. Namely, the reported unconventional interference effect is manifested only above a certain bias voltage applied on the normal lead. Secondly, the amplitude of the interfering path $N \to \mathrm{mol} \to S2 \to \mathrm{mol} \to S1 \to \mathrm{mol} \to S2 \to \mathrm{mol} \to N$ depicted in Fig. 3. of the main text is expected to be much smaller than the amplitude of the interfering path $N \to \mathrm{mol} \to S2 \to \mathrm{mol} \to N$, yet the resulting interference pattern in the Andreev current seem to be quite robust (see Fig. 4 in the main text). (The interfering path $N \to \mathrm{mol} \to S2 \to \mathrm{mol} \to S1 \to \mathrm{mol} \to S2 \to \mathrm{mol} \to N$ depicted in Fig. 3. of the main text involves four extra tunnelings between the leads and the central molecule compared to the interfering path $N \to \mathrm{mol} \to S2 \to \mathrm{mol} \to N$.)

The Andreev current can be evaluated using Eq. (4) of the main text. In this equation the lesser Green's function $G^<$ can be calculated within the Keldysh non-equilibrium framework using the Keldysh equation[2–6]:

$$G^< = G^R \Sigma^< G^A, \tag{S1}$$

where $G^R(E)$ $[G^A(E)]$ is the retarded [advanced] Green's function and $\Sigma^<(E) = \Sigma^<_{S1}(E) + \Sigma^<_{S2}(E) + \Sigma^<_N(E,V)$ contains the lesser self energies of the leads.

The differential conductance can be derived from Eq. (4) of the main text utilizing the relation given by Eq. (S1):

$$\frac{\mathrm{d}I_N}{\mathrm{d}eV} = -\frac{2e}{h}\mathrm{Re}\left\{\frac{\mathrm{d}}{\mathrm{d}eV}\int dE\ \mathrm{Tr}\left[\tau_3 W_N G^R\left(\Sigma^<_{S1} + \Sigma^<_{S2} + \Sigma^<_N(eV)\right)G^A\right]\right\} \tag{S2}$$

This expression can be further simplified by applying the derivation with respect to the bias voltage V on the integrand. Notice that only the self energy of the normal lead depends on eV. Hence

$$\frac{\mathrm{d}I_N}{\mathrm{d}eV} = -\frac{2e}{h}\mathrm{Re}\left\{\int dE\ \mathrm{Tr}\left[\tau_3 W_N G^R \frac{\mathrm{d}}{\mathrm{d}eV}\Sigma^<_N(E,eV) G^A\right]\right\} \tag{S3}$$

Furthermore, the lesser self energy $\Sigma^<_N(E,eV)$ depends on the bias voltage via the thermal occupation number. In the electron-hole space the lesser self energy can be given as[4]

$$\Sigma^<_N = \begin{pmatrix} f_e(\Sigma^R_{N,e} - \Sigma^A_{N,e}) & 0 \\ 0 & -f_h(\Sigma^R_{N,h} - \Sigma^A_{N,h}) \end{pmatrix} \tag{S4}$$

$$= \begin{pmatrix} f_e\left(\left(g^A_{N,e}\right)^{-1} - \left(g^R_{N,e}\right)^{-1}\right) & 0 \\ 0 & f_h\left(\left(g^A_{N,h}\right)^{-1} - \left(g^R_{N,h}\right)^{-1}\right) \end{pmatrix}, \tag{S5}$$

where $f_e = f(E - eV)$ $[f_h = f(E + eV)]$ is the thermal occupation number for the electrons [holes] given by the Fermi-distribution function and $\Sigma^R_{N,e}$ $[\Sigma^A_{N,e}]$ and $\Sigma^R_{N,h}$ $[\Sigma^A_{N,h}]$ are the retarded [advanced] self energies of the electron-like and hole-like particles in the normal lead, uncoupled from the rest of the system. Similarly, $g^R_{N,e/h}$ and $g^A_{N,e/h}$ stand for the retarded and advanced Green's functions of the electron/hole-like particles in the normal lead. To calculate the retarded and advanced self energies and Green's functions we

followed the numerical procedure described in Ref.[7]. Also, we assume the uncoupled leads to be in thermal equilibrium.

For simplicity we will consider the zero temperature limit in our calculations. Consequently, the derivative of the Fermi distribution function is the Dirac delta function and the integral in Eq. (S3) simplifies to

$$\begin{aligned}\frac{\mathrm{d}I_N}{\mathrm{d}V} = &-\frac{2e}{h}\mathrm{Re}\left\{\mathrm{Tr}\left[\tau_3 W_N G^R(eV)\begin{pmatrix}\left(g^A_{N,e}(eV)\right)^{-1}-\left(g^R_{N,e}(eV)\right)^{-1} & 0\\ 0 & 0\end{pmatrix}G^A(eV)\right]\right\}\\ &+\frac{2e}{h}\mathrm{Re}\left\{\mathrm{Tr}\left[\tau_3 W_N G^R(-eV)\begin{pmatrix}0 & 0\\ 0 & \left(g^A_{N,h}(-eV)\right)^{-1}-\left(g^R_{N,h}(-eV)\right)^{-1}\end{pmatrix}G^A(-eV)\right]\right\}\end{aligned} \quad . \tag{S6}$$

As we can see from Eq. (S6), the key element to calculate the differential conductance is the retarded and advanced Green's functions G^R and G^A. Eq. (S6) then can be directly used to calculate numerically the differential conductance in the studied three-terminal junctions.

To get further insight into the physics of the transport process we follow the logic of Ref.[8] to evaluate these Green's functions in terms of the Dyson's equation. Let us denote the retarded Green's function of the unified system of the two superconducting contacts and the central molecular core by g^R_{mol}. Then the retarded Green's function of the whole Andreev interferometer can be evaluated in terms of the Dyson's equation:

$$G^R = \begin{pmatrix}\left(g^R_{\mathrm{mol}}\right)^{-1} & -W_N^\dagger\\ -W_N & \left(g^R_N\right)^{-1}\end{pmatrix}^{-1}, \tag{S7}$$

where

$$g^R_N = \begin{pmatrix} g^R_{N,e} & 0\\ 0 & g^R_{N,h}\end{pmatrix} \tag{S8}$$

is the Green's function of the normal lead containing both the electron and hole-like components. Equation (S7) yields for the individual components of the Green's function:

$$G^R = \begin{pmatrix} G^R_{\mathrm{mol,mol}} & G^R_{\mathrm{mol,N}}\\ G^R_{\mathrm{N,mol}} & G^R_{\mathrm{N,N}}\end{pmatrix} = \begin{pmatrix} g^R_{\mathrm{mol}}\left(1-W_N^\dagger g^R_N W_N g^R_{\mathrm{mol}}\right)^{-1} & g^R_{\mathrm{mol}}\left(1-W_N^\dagger g^R_N W_N g^R_{\mathrm{mol}}\right)^{-1} W_N^\dagger g^R_N\\ g^R_N\left(1-W_N g^R_{\mathrm{mol}} W_N^\dagger g^R_N\right)^{-1} W_N g^R_{\mathrm{mol}} & g^R_N\left(1-W_N g^R_{\mathrm{mol}} W_N^\dagger g^R_N\right)^{-1}\end{pmatrix}. \tag{S9}$$

Considering the rules of the matrix multiplication, and that the only non-zero elements of the lesser self energy of Eq. (S5) are the block diagonal parts related to the leads, in order to evaluate the differential conductance (S6) it is enough to consider the $G^R_{\mathrm{mol},N}$ block of the retarded Green's function and the $G^A_{N,N}$ part of the advanced Green's function. According to the structure of Eq. (S9) one finds:

$$\begin{aligned} G^R_{N,N} &= g^R_N \sum_{n=0}^{\infty}\left(W_N g^R_{\mathrm{mol}} W_N^\dagger g^R_N\right)^n = g^R_N + g^R_N W_N g^R_{\mathrm{mol}} \sum_{n=0}^{\infty}\left(W_N g^R_{\mathrm{mol}} W_N^\dagger g^R_N\right)^n W_N^\dagger g^R_N\\ &= g^R_N + g^R_N W_N g^R_{\mathrm{mol}}\left(1-W_N^\dagger g^R_N W_N g^R_{\mathrm{mol}}\right)^{-1} W_N^\dagger g^R_N = g^R_N + g^R_N W_N G^R_{\mathrm{mol,mol}} W_N^\dagger g^R_N,\end{aligned} \tag{S10}$$

and

$$G^R_{\mathrm{mol},N} = G^R_{\mathrm{mol,mol}} W_N^\dagger g^R_N \,. \tag{S11}$$

We now return to the evaluation of the differential conductance given by Eq. (S6). For simplicity we continue our calculations focusing on the first (electron-like) part of Eq. (S6). (Due to the electron-hole symmetry of the Bogoliubov-de Gennes equations, the hole-like part would give the same result.) Inserting Eqs. (S10) and (S11) into Eq. (S6) yields:

$$\begin{aligned}\frac{\mathrm{d}I^e_N}{\mathrm{d}V} &= -\frac{2e}{h}\mathrm{Re}\left\{\mathrm{Tr}\left[\tau_3 W_N G^R_{\mathrm{mol},N}\begin{pmatrix}\left(g^A_{N,e}\right)^{-1}-\left(g^R_{N,e}\right)^{-1} & 0\\ 0 & 0\end{pmatrix}G^A_{N,N}\right]\right\}\\ &= -\frac{4e}{h}\mathrm{Im}\left\{\mathrm{Tr}\left[\tau_3 W_N G^R_{\mathrm{mol,mol}} W_N^\dagger\begin{pmatrix}\mathrm{Im}\left(g^R_{N,e}\right) & 0\\ 0 & 0\end{pmatrix}\left(1+W_N G^A_{\mathrm{mol,mol}} W_N^\dagger g^A_N\right)\right]\right\}\,.\end{aligned} \tag{S12}$$

In Eq. (S12) we applied the identity $g^R_{N,e}-g^A_{N,e} = 2\mathrm{i}\,\mathrm{Im}\left(g^R_{N,e}\right)$. For simplicity let's suppose we have only one Andreev bound state (ABS) formed in the superconductor – molecular core – superconductor (S-mol-S) junction described by the Green's function g^R_{mol}. In the presence of the normal lead, the ABS's starts to leak out via the normal lead resulting in the broadening of the ABS energy levels. Since our main interest are the transport properties close to the mid of the HOMO-LUMO gap, in the relevant energy regime we do not expect

any further bound states in $G^R_{\mathrm{mol,mol}}$ besides the ones corresponding to the ABS's. Thus, we might approximate $G^R_{\mathrm{mol,mol}}$ as:

$$G^R_{\mathrm{mol,mol}}(E) \approx \frac{|ABS\rangle\langle ABS|}{E - E_{ABS} + \mathrm{i}\Gamma_{ABS}}. \tag{S13}$$

Here the state $|ABS\rangle$ represents the wave function of the ABS in the molecule of energy E_{ABS}, and $\Gamma_{ABS} = \left\langle ABS \middle| W_N^\dagger \mathrm{Im}\left(g_N^R\right) W_N \middle| ABS\right\rangle$ is the level broadening originating from the escape rate of the particles through the normal lead.[8] The mathematical expression for Γ_{ABS} calculates the overlap between the ABS wave function and the self energy of the normal lead. Thus, Γ_{ABS} can be divided into two distinct terms, one related to the escape rate of the electron-like and the second one to the escape rate of the hole-like particles. Namely, $\Gamma_{ABS} = \Gamma_{ABS,e} + \Gamma_{ABS,h}$, where:

$$\Gamma_{ABS,e} = \left\langle ABS \middle| W_N^\dagger \begin{pmatrix} \mathrm{Im}\left(g^R_{N,e}(E_{ABS})\right) & 0 \\ 0 & 0 \end{pmatrix} W_N \middle| ABS \right\rangle , \tag{S14}$$

and

$$\Gamma_{ABS,h} = \left\langle ABS \middle| W_N^\dagger \begin{pmatrix} 0 & 0 \\ 0 & \mathrm{Im}\left(g^R_{N,h}(E_{ABS})\right) \end{pmatrix} W_N \middle| ABS \right\rangle . \tag{S15}$$

Using the (S13) expression of $G^R_{\mathrm{mol,mol}}$ and the invariance of the $\mathrm{Tr}(\dots)$ function against the cyclic permutation of its arguments one obtains for the differential conductance:

$$\begin{aligned} \frac{\mathrm{d}I_N^e}{\mathrm{d}V} \approx & -\frac{4e}{h}\mathrm{Im}\frac{\left\langle ABS \middle| W_N^\dagger \begin{pmatrix} \mathrm{Im}\left(g^R_{N,e}\right) & 0 \\ 0 & 0 \end{pmatrix} W_N \middle| ABS \right\rangle}{eV - E_{ABS} + \mathrm{i}\Gamma_{ABS}} \\ & -\frac{4e}{h}\mathrm{Im}\left\{ \frac{\left\langle ABS \middle| W_N^\dagger \begin{pmatrix} \mathrm{Im}\left(g^R_{N,e}\right) & 0 \\ 0 & 0 \end{pmatrix} W_N \middle| ABS \right\rangle}{eV - E_{ABS} + \mathrm{i}\Gamma_{ABS}} \frac{\left\langle ABS \middle| W_N^\dagger g_N^A \tau_3 W_N \middle| ABS \right\rangle}{eV - E_{ABS} - \mathrm{i}\Gamma_{ABS}} \right\} . \end{aligned} \tag{S16}$$

Now making use of the definition of the broadening parameters $\Gamma_{ABS,e}$ and $\Gamma_{ABS,h}$ we end up with the following expression for the differential conductance:

$$\frac{\mathrm{d}I_N^e}{\mathrm{d}V} \approx \frac{8e}{h} \frac{\Gamma_{ABS,e}\Gamma_{ABS,h}}{(eV - E_{ABS})^2 + \Gamma^2_{ABS}} . \tag{S17}$$

In the above expression we neglected the energy dependence of the Green's function of the normal lead in a Γ_{ABS} wide vicinity of the energy E_{ABS}. Accounting also for the hole-like part of the differential conductance (S6) gives an additional factor of two in the final result due to the electron-hole symmetry. Thus, the total differential conductance would be given by Eq. (7) of the main text. In case we have more than one ABS in the junction, the first term of Eq. (S16) would turn into a sum of Lorentzian resonances, while the second term evolves into a more complex mathematical expression:

$$-\sum_{p,q}\mathrm{Im}\left\{ \frac{\left\langle p \middle| W_N^\dagger \begin{pmatrix} \mathrm{Im}\left(g^R_{N,e}\right) & 0 \\ 0 & 0 \end{pmatrix} W_N \middle| q \right\rangle}{eV - E_p + \mathrm{i}\Gamma_{pp}} \frac{\left\langle q \middle| W_N^\dagger g_N^A \tau_3 W_N \middle| p \right\rangle}{eV - E_q - \mathrm{i}\Gamma_{qq}} \right\} = \mathrm{Im}\sum_{p,q} \frac{\Gamma_{pq,e}}{eV - E_p + \mathrm{i}\Gamma_{pp}} \frac{\Gamma_{qp,e} - \Gamma_{qp,h}}{eV - E_q - \mathrm{i}\Gamma_{qq}} \tag{S18}$$

where $|q\rangle$, E_q and Γ_q represents the wave function, the energy and the broadening of the qth ABS, and the quantities $\Gamma_{qp,e}$ and $\Gamma_{qp,h}$ are defined similarly to Eqs. (S14) and (S15), but the scalar product is taken between wave functions corresponding to different ABS's. Besides regular Lorentzian resonances [$p = q$ terms of Eq. (S18)] we see that the differential conductance is heavily influenced by the cross-talk of the individual ABS's. Mathematically the product of two fractions on the right hand side of Eq. (S18) can be rewritten to a sum

$$\frac{\Gamma_{pq,e}}{eV - E_p + \mathrm{i}\Gamma_{pp}} \frac{\Gamma_{qp,e} - \Gamma_{qp,h}}{eV - E_q - \mathrm{i}\Gamma_{qq}} = \frac{\lambda}{eV - E_p + \mathrm{i}\Gamma_{pp}} + \frac{\delta}{eV - E_q - \mathrm{i}\Gamma_{qq}} , \tag{S19}$$

where λ and δ are in general complex numbers. (Individually both of them have singularity at $eV = (\Gamma_{pp}E_q + \Gamma_{qq}E_p)/(\Gamma_{pp} + \Gamma_{qq})$, but these singularities cancel each other in the sum of the two fractions.) Consequently, the imaginary part of these fractions would differ from the regular Lorentzian function and the total differential conductance in the presence of multiple ABS's would be the sum of asymmetric Lorentzian resonances centered to the energies of the ABS's. The asymmetry in the resonances is a signature of the cross-talk between the ABS's.

S1.1 Resonant oscillation

As discussed in the main text, we try to explain the unconventional interference pattern by the interplay of the two paths depicted in Fig. 2 of the main text. However the amplitude $t_{9,22}$ (defined by Eq. (1) of the main text) might be expected to be much larger than the amplitude $t^{9}_{6,22}$ (defined by Eq. (5) of the main text) which would suppress the interference effect between these two interfering paths.

The physical picture behind the small magnitude of $t^{9}_{6,22}$ relative to $t_{9,22}$ is associated to the particle transfer between the two superconducting banks. The four tunneling processes between the molecular core and the superconducting electrodes significantly decreases the magnitude of the interfering amplitude $t^{9}_{6,22}$. On the other hand, a resonant oscillation realized by the ABSs overwrites this physical picture. In this case the charge transport between the superconducting banks becomes resonantly amplified via the ABS and thus the amplitudes $t^{9}_{6,22}$ and $t_{9,22}$ becomes comparable. In summary, for energies close enough to the energy of an ABS the differential conductance shows an interference effect due to the resonant amplification of the interfering amplitude $t^{9}_{6,22}$, while for other energies the interference would be suppressed.

S1.2 Density of states

In this subsection we give the technical details to calculate the density of states of the three-terminal molecular junction, which can be used to physically interpret the numerical results obtained by Eqs. (4) of the main text and by Eq. (S6). We calculate the density of statesρ from the equilibrium Green's function of the three-terminal molecular junction labeled by $G^{R}_{\mathrm{mol,mol}}$ in the calculations above. To be precise, $G^{R}_{\mathrm{mol,mol}}$ labels only that block of the whole Green's function which contains only the molecular degrees of freedom. Then the density of states can be defined as:

$$\rho(E) = -\frac{1}{\pi}\mathrm{Tr}\left[\mathrm{Im}\left(G^{R}_{\mathrm{mol,mol}}(E)\right)\right] . \tag{S20}$$

As for the differential conductance, $G^{R}_{\mathrm{mol,mol}}$ can be calculated via the Dyson's equation (S7) which is evaluated using the Eötvös Quantum Utilities (EQuUs) [1] software package.

S2 The tight-binding model of the molecular junctions

To describe the electrical transport processes in the studied molecular junctions we use a nearest neighbor tight binding model catching the dynamics of the p_z electrons of the molecular core.

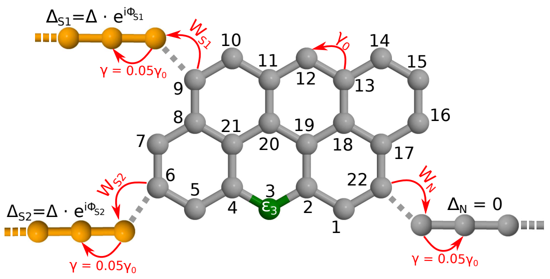

Fig. 1 The tight binding model of the Anthanthrene molecule attached to two superconductive and one normal lead. The sites in the molecular core are labeled by primed and unprimed numbers, while the hopping amplitude between the sites are characterized by a single number. The normal and superconducting contacts are modeled by a one-dimensional conductive channels.

The tight binding parameters describing the molecular core are chosen following the philosophy in Refs.[9,10], where the aim is to highlight the role of connectivity in determining the transport properties of these molecular cores. For this reason, the hopping integrals $\gamma_{ii'} = \gamma_0$ are set to unity and the on-site energies ε_i are set to zero. With other words, the unit of energy is the hopping integral and the site energy is the energy origin. This means that the Hamiltonian of the molecule is simply a connectivity matrix and therefore all predicted effects are a result of connectivity alone. The normal and superconducting contacts are modeled by a one-dimensional tight-binding chain. The transport properties of the junction have a weak dependence on the actual physical parameters of the leads as far as the leads remains metallic in the studied energy regime. Thus, we chose the physical parameters of the leads to increase the density of states in the leads and have the bandwidth of the conductive larger than the studied energy regime. In particular, we set the hopping amplitude in the contacts to $0.05\gamma_0$ and the on-site energy parameter to 0. The superconducting contacts are modeled by an s-type superconducting pair potential $\Delta = 0.001\gamma_0$. (The pairing potential is zero anywhere else in the system.) In the particular case the tight binding model of the Anthanthrene molecule connected to the superconducting and normal electrodes is shown in Fig. 1. Remarkably, as demonstrated in Refs.[9,10], this approach yields the experimentally-measured conductance ratios of a range of PAHs.

Finally, as we explained in the main text, we tuned the transport properties of the molecular core by an inserting a substitutional heteroatom into the molecular core. According to Ref.[11], the presence of the heteroatom have a strong influence on the inner quantum interference effects in the molecular core, even new conductive channels may open up in the molecular core. In our theoretical model

we account for the presence of a substitutional heteroatom by a modified on-site energy on a specific site in the molecule.

S3 Comparison of the local density of states on two molecular sites

As shown in Fig.8(c) and (d) of the the main text, which is reproduced below in Fig. 2, the local density of states (LDOS) is suppressed for electron-like quasiparticles and enhanced for hole-like quasiparticles on molecular site 22 (for the numbering of the molecular sites, see Fig. 1). We have calculated the LDOS for the other sites of the molecular core as well and found that due to QI the LDOS of the electron and hole quasiparticles is different on each site. In particular, it can happen that, in contrast to Fig. 2, the electron LDOS is larger than the hole LDOS. An example shown in Fig. 3, where this asymmetry of LDOS can be clearly seen.

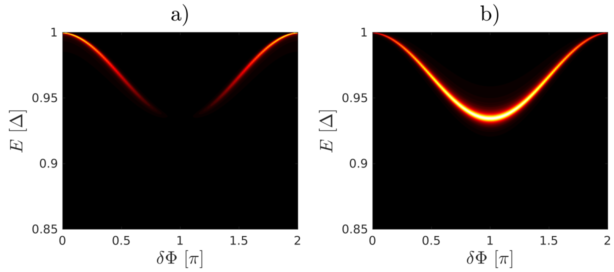

Fig. 2 The LDOS for electron (a) and hole (b) quasiparticles as a function of $\delta\Phi$ on molecular site 22 of the Andreev interferometer shown in Fig.6(c) of the main text and in Fig. 1. In these calculations $\varepsilon_3 = -0.50\gamma_0$.

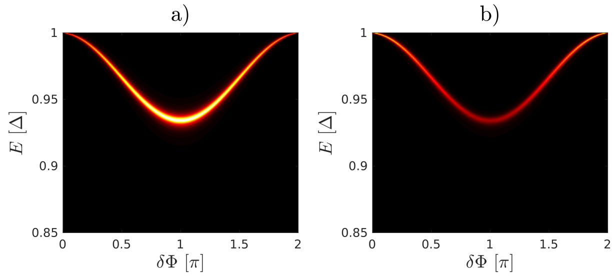

Fig. 3 The LDOS for electron (a) and hole (b) quasiparticles as a function of $\delta\Phi$ on molecular site 8 of the Andreev interferometer shown in Fig.6(c) of the main text and in Fig. 1. In these calculations we used $\varepsilon_3 = -0.50\gamma_0$.

As mentioned in the "Conclusions and Outlook" section of the main text, by attaching normal leads $N1$ and $N2$ to molecular sites 8 and 22 and may enhance the non-local Andreev reflection $N1 \to N2$ with respect to the local Andreev reflection $N1 \to N1$.

Notes and references

1 *Eötvös Quantum Utilities*, http://eqt.elte.hu/EQuUs/html/.
2 C. J. Bolech and T. Giamarchi, *Phys. Rev. B*, 2005, **71**, 024517.
3 S.-T. Wu and S. Yip, *Phys. Rev. B*, 2004, **70**, 104511.
4 A. Cresti, R. Farchioni, G. Grosso and G. P. Parravicini, *Phys. Rev. B*, 2003, **68**, 075306.
5 V.-N. Do, *Advances in Natural Sciences: Nanoscience and Nanotechnology*, 2014, **5**, 033001.
6 M. G. Pala, M. Governale and J. König, *New Journal of Physics*, 2007, **9**, 278–278.
7 I. Rungger and S. Sanvito, *Phys. Rev. B*, 2008, **78**, 035407.
8 N. R. Claughton, M. Leadbeater and C. J. Lambert, *Journal of Physics: Condensed Matter*, 1995, **7**, 8757–8784.
9 Y. Geng, S. Sangtarash, C. Huang, H. Sadeghi, Y. Fu, W. Hong, T. Wandlowski, S. Decurtins, C. J. Lambert and S.-X. Liu, *Journal of the American Chemical Society*, 2015, **137**, 4469–4476.
10 S. Sangtarash, C. Huang, H. Sadeghi, G. Sorohhov, J. Hauser, T. Wandlowski, W. Hong, S. Decurtins, S.-X. Liu and C. J. Lambert, *Journal of the American Chemical Society*, 2015, **137**, 11425–11431.
11 S. Sangtarash, H. Sadeghi and C. J. Lambert, *Nanoscale*, 2016, **8**, 13199–13205.

[a] *Dept. of Physics of Complex Systems, Eötvös Loránd University, Budapest, Pázmány P. s. 1/A, Hungary; E-mail: rakytap@caesar.elte.hu*
